# Interhemispheric Connections between the Primary Visual Cortical Areas via the Anterior Commissure in Human Callosal Agenesis

**DOI:** 10.3389/fnsys.2016.00101

**Published:** 2016-12-26

**Authors:** Nathalie van Meer, Anne C. Houtman, Peter Van Schuerbeek, Tim Vanderhasselt, Chantal Milleret, Marcel P. ten Tusscher

**Affiliations:** ^1^Department of Ophthalmology, Universitair Ziekenhuis, Vrije Universiteit BrusselBrussels, Belgium; ^2^Department of Radiology, Universitair Ziekenhuis, Vrije Universiteit BrusselBrussels, Belgium; ^3^Brain Rhythms and Neural Coding of Memory, Center for Interdisciplinary Research in Biology, Collège de France, Centre National de la Recherche Scientifique, Institut National de la Santé et de la Recherche Médicale, Paris Sciences et Lettres Research UniversityParis, France

**Keywords:** corpus callosum agenesis, visual interhemispheric communication, anterior commissure, diffusion tensor imaging (DTI), primary visual cortex, binocularity

## Abstract

**Aim:** In humans, images in the median plane of the head either fall on both nasal hemi-retinas or on both temporal hemi-retinas. Interhemispheric connections allow cortical cells to have receptive fields on opposite sides. The major interhemispheric connection, the corpus callosum, is implicated in central stereopsis and disparity detection in front of the fixation plane. Yet individuals with agenesis of the corpus callosum may show normal stereopsis and disparity vergence. We set out to study a possible interhemispheric connection between primary visual cortical areas via the anterior commissure to explain this inconsistency because of the major role of these cortical areas in elaborating 3D visual perception.

**Methods:** MRI, DTI and tractography of the brain of a 53-year old man with complete callosal agenesis and normal binocular single vision was undertaken. Tractography seed points were placed in both the right and the left V1 and V2. Nine individuals with both an intact corpus callosum and normal binocularity served as controls.

**Results:** Interhemispheric tracts through the anterior commissure linking both V1 and V2 visual cortical areas bilaterally were indeed shown in the subject with callosal agenesis. All other individuals showed interhemispheric visual connections through the corpus callosum only.

**Conclusion:** Callosal agenesis may result in anomalous interhemispheric connections of the primary visual areas via the anterior commissure. It is proposed here that these connections form as alternative to the normal callosal pathway and may participate in binocularity.

## Introduction

The hallmark of human binocular vision is partial decussation in the optic chiasm. Ganglion cells from the nasal half of each retina cross over to the contralateral hemisphere whereas ganglion cells from each temporal half remain on the ipsilateral side (Hubel, 1988). It allows two retinal ganglion cells, one from each eye, but representing the same part of the visual field, to pair up and finally drive a binocular neuron in layer IV-B of the primary visual cortex. Hemi-decussation implies that the visual field is divided into a left- and a right sided hemifield.

Objects in the median plane of the head either fall on both nasal halves of the retinae (images beyond the fixation point), or fall on both temporal halves of the retinas (images nearer to the fixation point). So, in order to detect binocular disparity of objects located on the visual midline, some cortical cells must have receptive fields in opposite hemi-retinas, thus in heteronymous hemifields. A less than perfect separation between crossed and uncrossed ganglion cells in the midline of the retina and the corpus callosum both serve to this end (Olavarria, 2001).

The midline of the retina, indeed, is not a sharp demarcation between crossed and uncrossed ganglion cells. In primates and other mammals (in particular with frontal vision), a small area around the retinal vertical midline, called the “naso-temporal region of overlap,” contains both crossed and uncrossed ganglion cells (Fukuda et al., 1989). This likely contributes to detect binocular disparity at the level of primary visual cortex, in particular for fine stereopsis. The contribution of the corpus callosum is twofold. The retinal midline from each eye relays visual information to both primary visual cortices and allows linking loci with retinotopic correspondence or adjacent loci through the corpus callosum (Poggio and Poggio, 1984; Innocenti, 1986). Additionally, more laterally placed retinal ganglion cells within the temporal retina relay visual information via the ipsilateral primary visual cortex, then through the corpus callosum and finally within the contralateral primary visual cortex. Here callosal recipient neurons thus display visual receptive fields located ipsilateral to the recipient visual cortex. These same neurons are binocular, receiving also afferents from the ipsilateral direct retino-geniculo-cortical projection. This provides a second receptive field but this time located within the contralateral visual hemifield for these same callosal recipient neurons (Milleret et al., 2005). The resultant disparities are mostly of the crossed type. Thus, the callosal pathway is thought to play a role in contributing not only to cortical binocularity but also to depth and disparity detection in front of the fixation plane (Aboitiz and Montiel, 2003). Note that, altogether, both the naso-temporal retinal overlap and the corpus callosum thus contribute to unify both visual hemifields in a single scene.

The corpus callosum is the largest commissure of the human brain containing about 200 million fibers of various diameters. Phylogenetically, it is not the oldest commissure in the brain. The anterior commissure, which is about 10 times smaller containing only 3.5 million fibers, appeared first during evolution (Innocenti, 1986). Even in the brain of non-placental mammals like marsupials there is no callosal connection. Instead, the primary form of interhemispheric communication is through an enlarged anterior commissure (Pietrasanta et al., 2013) which has been shown to be functionally equivalent to the corpus callosum (Guénot, 1998). Of interest, the embryologic development of the commissures in humans respects this phylogenetic order. Fibers within the anterior commissure develop first from the 8th to 11th week of pregnancy (crossing the midline at ~9 weeks). The corpus callosum develops later on, from anterior to posterior, with the first fibers crossing the midline not before the 13th week of pregnancy. Crossing in the posterior part, the splenium, is completed around the 20th to 22nd week of gestation. Finally, both mature until myelination is completed around puberty (Raybaud, 2010; Yousefi, 2012 for reviews).

In the posterior part of the human corpus callosum, the splenium, two parts are distinguished. The posterior part interconnects the posterior hemi-cortices including the primary and secondary visual areas. The anterior part of the splenium predominantly connects the parietal and temporal areas (Ebner, 1969). Both parts, but especially the posterior part, are implicated in elaborating visual perception. Two parts have also been recognized in the anterior commissure: an anterior limb which forms an open “U” and a posterior limb which makes a flattened “M” when viewed in the axial plane. The anterior part, which is the oldest and the smallest one, connects the olfactory bulbs and the inferior posterior orbital gyri; a small number of axons also cross the mid-sagittal plane that is believed to reach territories other than the temporal cortex. In contrast, the posterior part, which forms the major and neocortical portion of the anterior commissure, travels within the basal parts of the putamen, the caudate nucleus and below the anterior border of the globus pallidus into the temporal cortex. It also projects to the amygdala, the temporal pole and the parahippocampal, inferior temporal and fusiform gyri. The anterior commissure thus contributes to various functions such as olfaction, memory, emotion, speech, hearing and sexual behavior. Of interest here, a few short fibers from this posterior limb of the anterior commissure have been described in relation to the occipital lobe: some of these fibers seem to project into it while others seem to arise from it (as well as from the precentral gyrus and the central fissure) (Di Virgilio et al., 1999; Catani et al., 2002; Mitchell et al., 2002; Patel et al., 2010). This connection with the occipital lobe has never been described systematically among the subjects under study (e.g., Jellison et al., 2004; Patel et al., 2010) or has been found to link both hemispheres. Nor were occipital visual cortical areas involved, identified (Yousefi, 2012 for review). Functionally, only few studies aimed at analyzing the role of the anterior commissure in transferring visual information from one hemisphere to the other. Gazzaniga and LeDoux (1978) identified some visual interhemispheric transfer after section of the corpus callosum, using rather complex visual stimuli such as pictorial stimuli, but the implication of the anterior commissure was just hypothetical (Risse et al., 1978). A more recent study also hypothesized a potential role of the anterior commissure in linking vision, action and attention (Winter and Frantz, 2014). Further studies are clearly needed to clarify the role of the anterior commissure in elaborating visual perception in the healthy individual.

The role of the anterior commissure seems compensatory in various pathological cases. In kittens, callosotomy during the sensitive period causes strabismus and loss of cortical binocularity; but after this period, motor fusion was found to be normal (Smith, 1902; Nelson and Lende, 1965). In humans, the corpus callosum may be absent at birth. This may be partial or complete agenesis, in isolation or in association with other neurological conditions. Individuals born with agenesis of the corpus callosum do not show the complete split-brain syndrome like patients who have undergone surgical callosotomy or suffered callosal damage in adulthood (Lassonde et al., 1991). Strabismus may also, but not necessarily, occur in patients with callosal dysgenesis. Goyal et al. (2010) showed strabismus in 46% of patients with partial agenesis. Patients often show residual interhemispheric communication. Interhemispheric anomalous rewiring through the commissures has been shown with preserved crossed transfer of complex tactile function between hemispheres (Tovar-Moll et al., 2014).

We speculated here that anomalous interhemispheric communication through the anterior commissure linking primary visual cortical areas may also explain the preservation of binocularity in cases of callosal agenesis with normal stereopsis and motor fusion. Diffusion tensor imaging and tractography were used to compare interhemispheric tracts between an individual with complete agenesis of the CC and intact binocular vision, and controls with normal binocularity and a normal corpus callosum.

## Materials and methods

A 53-year old man, with a known callosal agenesis was investigated. He is an otherwise healthy individual without medical history. He had a corrected visual acuity of 20/20 in both eyes and normal stereopsis. Assessment of binocularity revealed: straight eyes with a slight exophoria; which is a normal finding. Bagolini striated glasses, used for the detection of micro-strabismus, showed a symmetrical cross response (which means that there is not the slightest form of strabismus; no micro-strabismus). Stereoacuity (TNO Random dot stereo-test, a random-dot stereotest presented in an anaglyphic format) was 40 arcs (normal: less than 60 arcs). Motor fusion was within the normal range for near (33 cm: 14 prism diopters base inward to 18 prism diopters base outward) and for distance (5 m: 6 prism diopters base inward to 18 prism diopters base outward). So, in essence normal binocular vision and straight eyes in an individual with complete agenesis of the corpus callosum was encountered here.

Nine volunteer participants with no history of ophthalmologic- or neurologic disease served additionally as controls (mean age, 32 years; range, 23–52). All but one subject had a corrected visual acuity of 20/20 or better in both eyes, with normal binocular vision (stereopsis of at least 60”). The visual characteristics of all subjects are summarized in Table 1.

**Table 1:** **Visual perception characteristics of all individuals studied**.

	**Sex**	**Age**	**Vare**	**Vale**	**Sta**
**Subjects**					
RTM 26	M	28	20/20	20/20	60
RTM 28	F	23	20/20	20/20	60
RTM 29	M	27	20/20	20/20	40
RTM 30	F	34	20/20	20/20	60
RTM 32	F	24	20/20	20/20	40
RTM 33	F	24	20/20	20/20	120
RTM 35	F	30	20/20	20/20	60
RTM 38	M	50	20/20	20/20	60
RTM 39	F	52	20/20	20/20	60
CC ag.	M	53	20/20	20/20	40

*All RTM's are healthy controls with an intact corpus callosum. The individual with callosal agenesis is indicated as CC ag. Vare, visual acuity right eye; vale, visual acuity left eye; sta, stereo acuity in seconds of arc; determined with a random dot stereotest (TNO); M, male; F, female*.

Full eye examinations of each subject (with callosal agenesis and controls) were performed by an ophthalmologist to exclude eye disease. Participants had no contraindications for MRI and informed consent was obtained from all participants before data collection. The present study adhered to the tenets of the Declaration of Helsinki. Medical ethical approval was obtained from the institutional review board.

Imaging of the participant with callosal agenesis was done at a 3T GE MRI scanner (MR 750w Discovery, USA). The scan protocol consisted of a 3D GR scan (FOV: 240 × 240 × 163.2 mm, resolution: 0.47 × 0.47 × 0.6 mm, TI = 450 ms, TR = 8.56 ms, TE = 3.36 ms, flip angle = 12°) and a DTI scan (FOV: 200 × 200 × 163.2 mm, resolution: 0.78 × 0.78 × 2.5 mm, TR = 8970 ms, TE = 80 ms, 30 diffusion directions with *b* = 1000 and 6 scans with *b* = 0). Imaging of all 9 normal subjects was done at a 3T Philips MRI-scanner (Achieva, Philips, The Netherlands). A high resolution structural scan was made with a 3D TFE sequence (FOV: 240 × 240 × 120 m, resolution: 1 × 1 × 2, TI = 10 ms, TR = 12 ms, TE = 3.74 ms). The DTI scan was done using a DwiSE sequence (FOV: 224 × 224 × 120 mm, resolution: 2 × 2 × 2, TR = 7337 ms, TE = 83 ms, 31 diffusion directions with *b* = 800 and 1 *b* = 0 image). The different settings between the individuals with callosal agenesis and controls are based on the use of a different scanner. Therefore, a difference in diffusion between both protocols can't be excluded. This possible difference, however, cannot explain the different results.

For each subject the structural scan was processed in FreeSurfer using the default processing settings. Firstly, the brain was extracted from the structural scan. Secondly, the neuroanatomical subareas as defined in the Talairach atlas were located in each individual brain, taking the individual anatomy into account. From the labeled regions of interest (ROIs), the left and right V1 and V2 labeled areas were selected, for later use in the DTI analysis.

Prior to the actual DTI analysis, the DTI scans were corrected for movement and eddy currents in FSL (Jenkinson and Smith, 2001; Smith et al., 2004). Secondly, the anatomical scan was registered to the b0-DTI scan and the determined affine registration parameters were used to register the V1 and V2 ROIs to the DTI scans. On the anatomical scan, a spherical ROI was drawn in the anterior commissure (AC) In order to visualize the majority of tracts going through the anterior commissure in the individual with agenesis of the corpus callosum, a separate analysis was undertaken in which the anterior commissure was used as a single ROI without using way points or end points. The final analysis was done in DSI Studio using the generalized q-sampling (GQI) algorithm (Yeh et al., 2010) with a diffusion sampling length ratio of 1.25. The fiber tracking analyses were done with a deterministic fiber tracking algorithm (Yeh et al., 2014) with a qa threshold automatic determined by DSI studio, an angular threshold of 50°, a step size of 0.10mm and a total of 10,000 seeds randomly placed in the seed area. Several fiber tracking analyses were done varying in selected ROI used as seed area and a selected second ROI area used as the area through which the fibers had to pass or to end, to end up with a specific fiber bundle. After the fiber tracking done by the software, the resulting fibers were inspected visually and erroneous fibers were manually deleted. These fibers resulted from the small interhemispherical distance between the medial areas of the visual cortices on both sides. Due to the limited resolution of the DTI technique, tracts were reconstructed directly crossing over to the other side. The number of these fibers differed with every analysis. In succession, we tracked the fibers in the whole brain, from V1 left to V1 right, from V1 left to V2 right, from V2 left to V1 right, from V2 left to V2 right, from V1 right to V1 left, from V1 right to V2 left, from V2 right to V1 left, from V2 right to V2 left, from V1 left to AC, from V2 left to AC, from V1 right to AC and from V2 right to AC. Note that, for convenience, we have considered both V1 and V2 as “primary” visual cortical areas, although V2 is generally considered as the first area of the ventral stream. As control experiment a DTI analysis was undertaken in which the posterior commissure was used as directional ROI instead of the anterior commissure.

## Results

**Figure 1:**
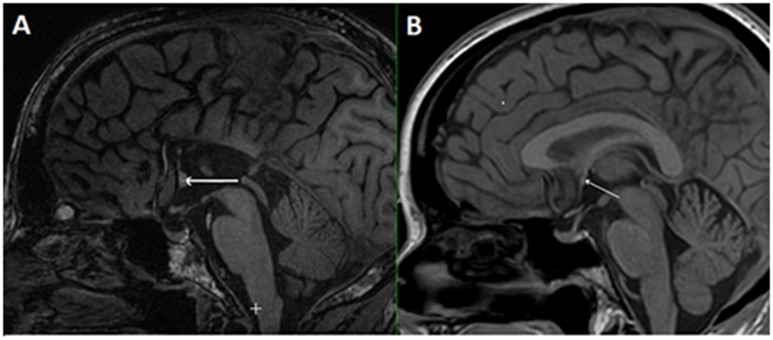
**Mid-sagittal plane MRI section of the individual with callosal agenesis (A)**. The arrow indicates the enlarged anterior commissure (diameter 5.2 mm) as compared to a control individual **(B)** with a corpus callosum and a normal anterior commissure with a diameter of 1.8 mm (arrow).

**Figure 2:**
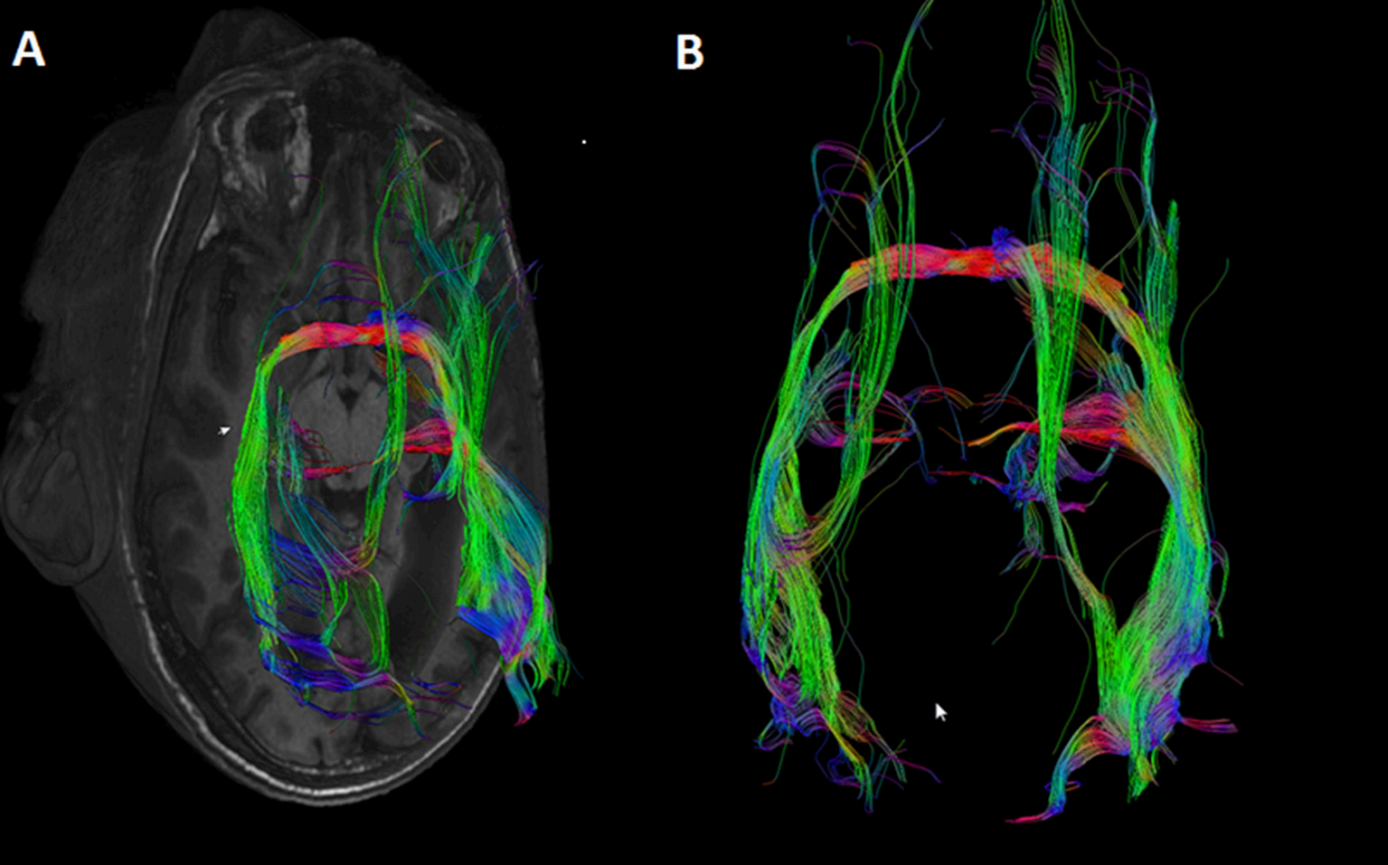
**DTI tracts going through the anterior commissure in the individual in which the corpus callosum was missing**. Seeds were placed in the anterior commissure without indicating any other region of interest. Large tracts project to the occipital cortical tips on both sides. **(A)** Axial MRI section with superimposed DTI tracts. **(B)** DTI tracts from superior (anterior side of the head on top of the image).

In the case of callosal agenesis, MRI showed a complete absence of the corpus callosum with parallel, enlarged lateral ventricles. The size of the anterior commissure in the individual with callosal agenesis, diameter 5.2 mm (Figure 1A) was clearly much larger than normal (diameter 1.8 mm) (Figure 1B). Abnormal interhemispheric fibers appeared with tractography. In the analysis with the anterior commissure as single ROI massive tracts running toward the occipital cortex along the border of the lateral ventricles appeared (Figure 2).

**Figure 3:**
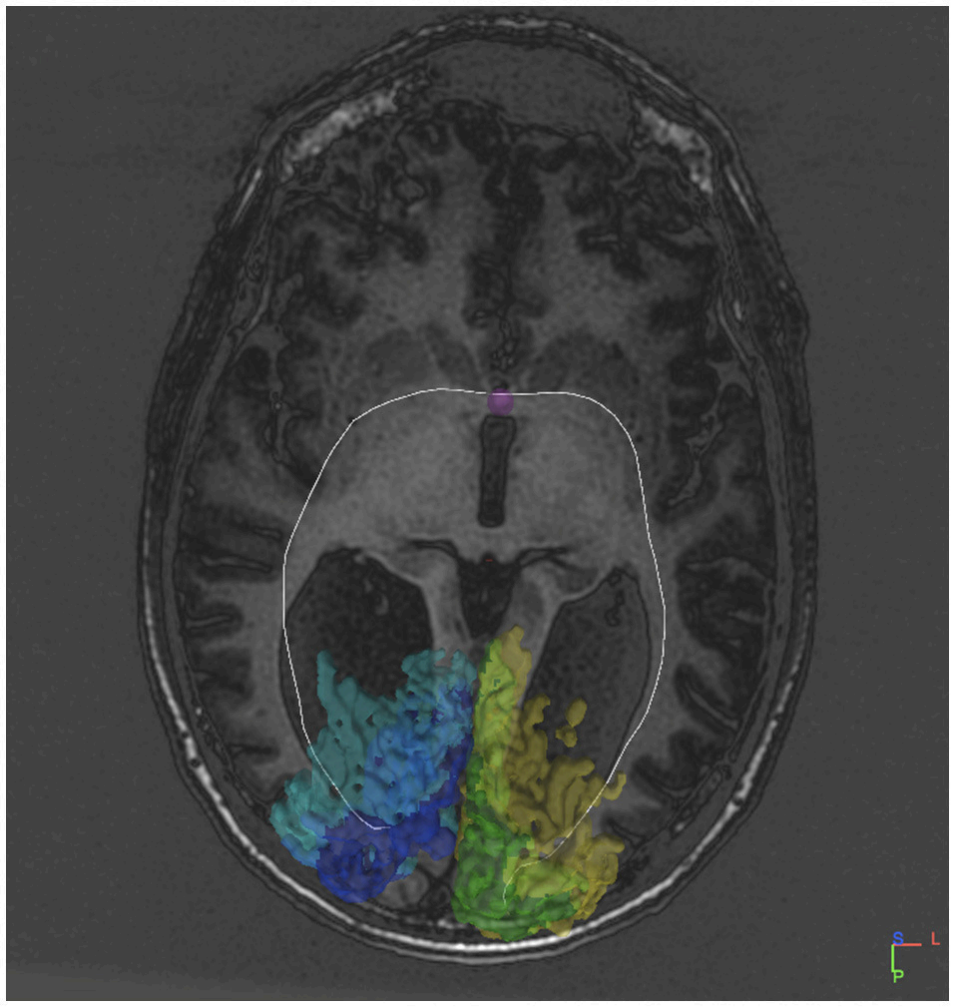
**DTI tract connecting V1V2 on both sides via the anterior commissure in an individual without a corpus callosum**.

Indeed, several (2–8) fibers appeared to project to the posterior part of the anterior commissure both from the right V1 and V2, and from the left V1 and V2 (Table 2). The primary visual cortices were thus connected bilaterally via the anterior commissure instead of being interconnected by the corpus callosum. One entire tract was identified from the left V2 to the right V1 (Figure 3). From the right visual cortical areas an entire tract was not visualized. In fact several tracts starting from the right (5) and left (14) V1 and V2 were identified up to the anterior commissure but could not be traced to the occipital areas on the other side.

**Table 2:** **Shows the number of tracts identified with DTI in 9 normal controls (RTM's) and in one subject with agenesis of the corpus callosum (Cal. Agen.)**.

	**RTM 26**	**RTM 28**	**RTM 29**	**RTM 30**	**RTM 32**	**RTM 33**	**RTM 35**	**RTM 38**	**RTM 39**	**CAL. Agen**.
V1L-V1R	0	11	3	15	67	3	2	38	0	0
V1L-V2R	4	9	3	64	115	1	11	3	0	0
V2L-V1R	10	12	4	27	13	3	0	17	10	1^*^
V2L-V2R	31	6	2	108	46	4	0	6	6	0
V1R-V1L	0	7	3	15	104	3	2	38	0	0
V1R-V2L	0	43	2	50	6	25	20	42	6	0
V2R-V1L	16	4	8	53	52	0	4	0	1	0
V2R-V2L	14	13	15	165	43	14	15	7	4	0
VIL-AC	0	0	0	0	0	0	0	0	0	6
V2L-AC	0	0	0	0	0	0	0	0	0	8
VIR-AC	0	0	0	0	0	0	0	0	0	3
V2R-AC	0	0	0	0	0	0	0	0	0	2
VC-PC	0	0	0	0	0	0	0	0	0	0

Seeds from which tracts are identified were placed in V1 or V2. Interhemispheric fibers which connect visual cortical areas on the right with visual cortical areas on the left are shown in the first eight rows. These fibers course through the corpus callosum except for the one fiber indicated with ^*^. This fiber courses via the anterior commissure. The four rows in the middle show fibers from the primary visual areas which project to the anterior commissure. The last row shows fibers from the primary visual areas toward the posterior commissure. AC, Anterior commissure; PC, Posterior commissure; VC, visual cortex; R, right; L, left.

**Figure 4:**
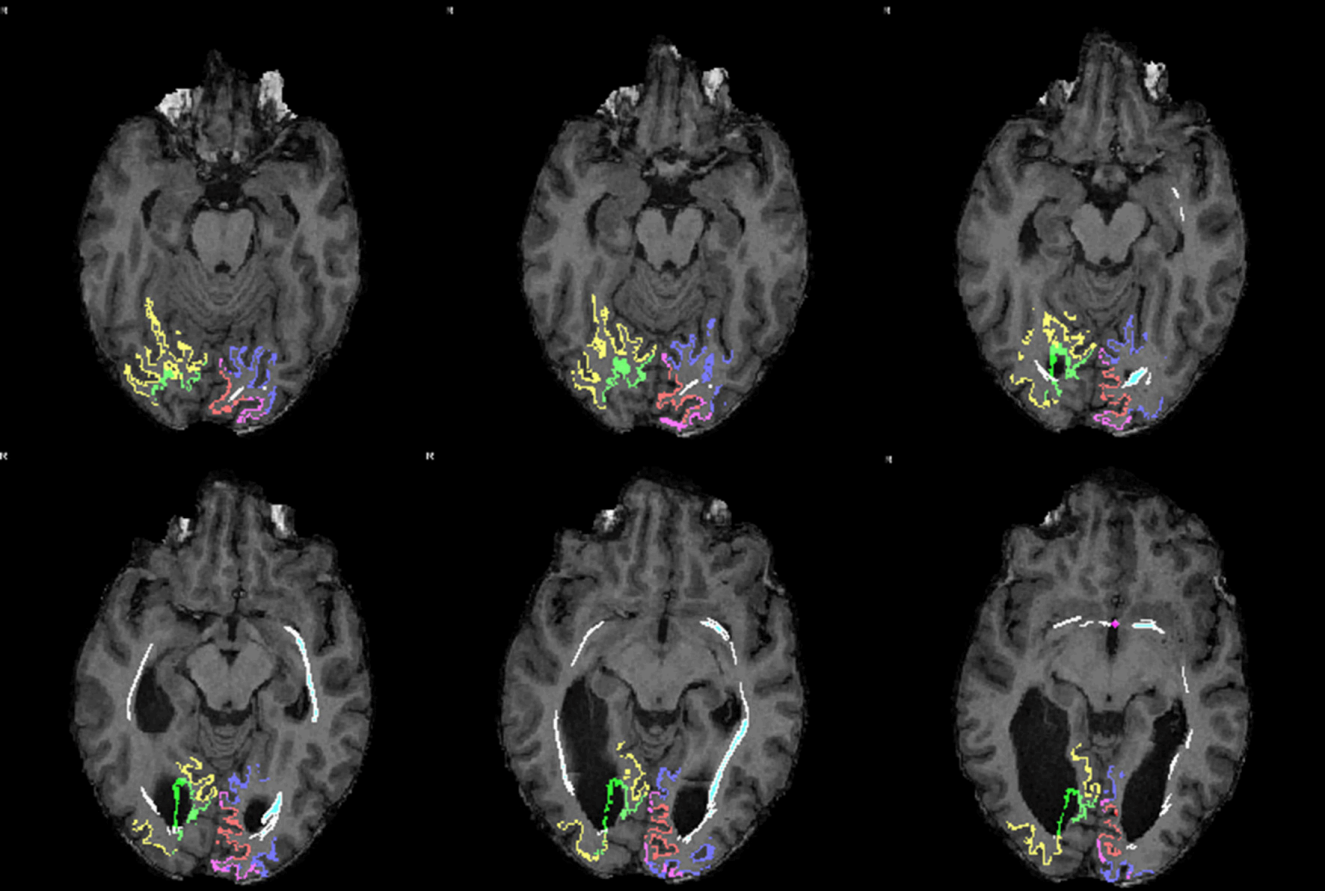
**Sequential mid-horizontal axial MRI/DTI sections showing V1 (green/pink), V2 (yellow/purple) and a (white) DTI tract connecting the primary cortical visual areas bilaterally via the anterior commissure in the individual without corpus callosum**. Seeds were placed in the primary visual areas with the anterior commissure as region of interest. It shows the fiber tract association with the primary visual areas on both sides.

The association between the tract which could be reconstructed between bilateral visual areas via the anterior commissure and the delineated visual areas was studied in individual axial MRI slices (Figure 4). Due to the limited anatomical accuracy of the DTI technique it was difficult to verify if tracts arose from the area alongside the V1/V2 border, i.e., the vertical midline representation and fovea. But tracts did clearly show a dorsal and a ventral occipital distribution.

**Figure 5:**
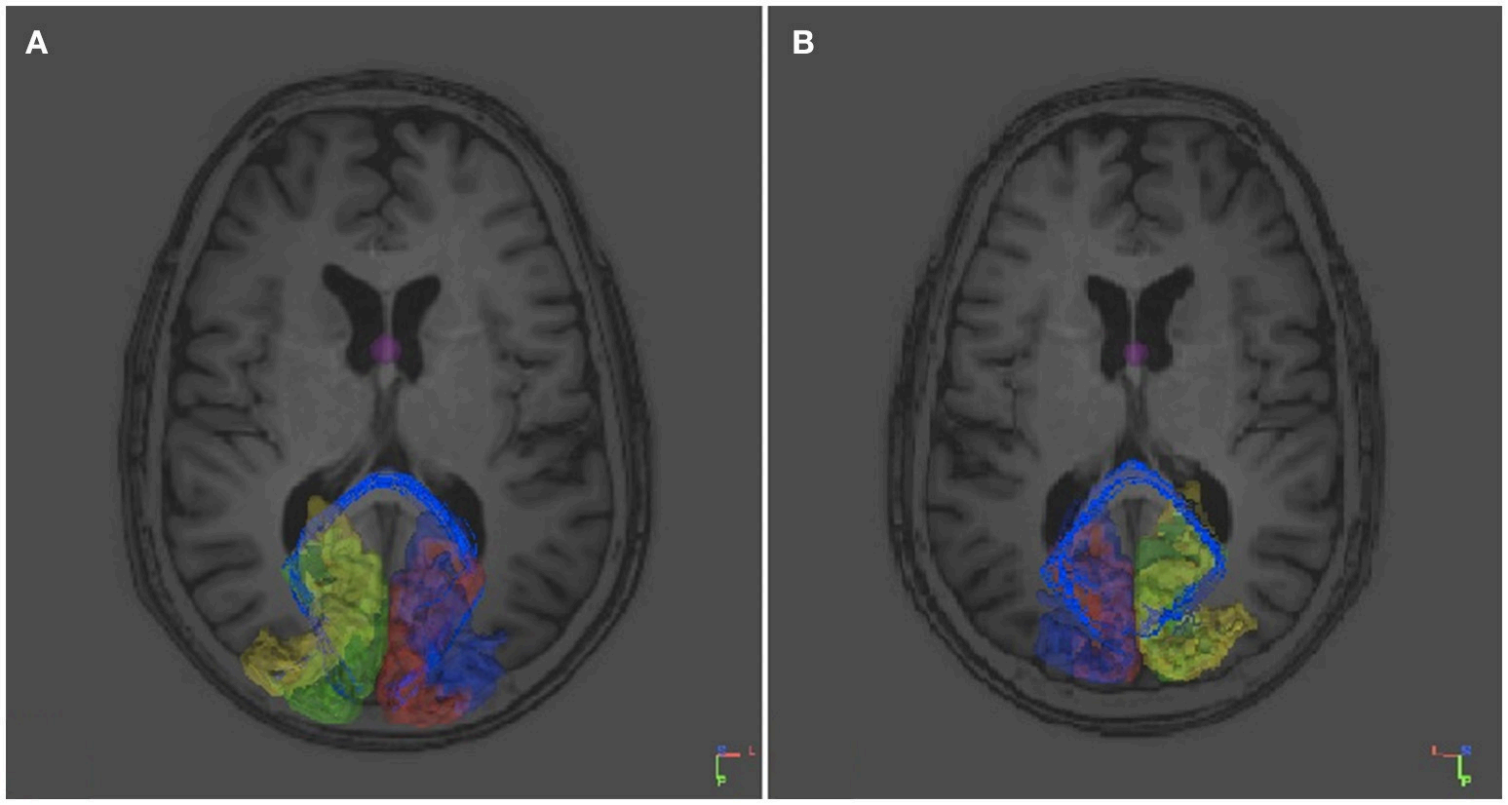
**DTI tracts connecting the right and left V1 and V2 in a control subject (RTM 30)**. In the left occipital cortex V1 is shown in red and V2 in blue. In the right occipital cortex V1 is green and V2 yellow. DTI tracts connecting both visual areas via the corpus callosum are shown in blue. There were no tracts that course through the anterior commissure. **(A)** An axial plane MRI image is shown from below. Tracts seem to aim for the border area between V1 and V2. **(B)** An axial plane image shows the same tracts from above.

In contrast, not surprisingly, in every individual used as control numerous fibers appeared to project contralaterally through the splenium of the corpus callosum both from the right V1 and V2 and from the left V1 and V2 (Figure 5). Not a single control individual displayed fibers from the left or the right V1 and V2 areas crossing over via the anterior commissure.

In the control experiment in which the posterior commissure, instead of the anterior commissure, was taken as ROI, both in the individual with callosal agenesis and in control individuals, not a single tract could be reconstructed from the visual areas toward the posterior commissure.

## Discussion

A man with known callosal agenesis and normal stereopsis appears here to have reciprocal interhemispheric connections between the visual cortical areas V1 and V2 through the anterior commissure. In contrast, an equivalent tractography analysis in 9 individuals with an intact corpus callosum showed interhemispheric connectivity of the occipital lobes only through the corpus callosum.

In 1970, Mitchell and Blakemore hypothesized that there is a role for the corpus callosum in central vision and depth perception. When two images fall on opposite sides of the two foveas, i.e., bi-temporal or bi-nasal disparity, the information of the two hemispheres has to be combined in order to detect disparity which is used for vergence and depth perception. It was put to the test in a subject who had his corpus callosum cut, to relieve epilepsy, and they found the patient to be stereo-blind in the central visual field.

The likely role of the corpus callosum in oculomotor fusion has also been shown in kittens which were reared with strabismus. Bilateral connections of the primary visual cortices through the corpus callosum were found to be asymmetrical in cats with induced early unilateral strabismus. Callosal terminals were found to be expanded in the primary visual cortex ipsilateral to the deviated (convergent) eye and almost normal in the contralateral hemisphere (Milleret and Houzel, 2001; Bui Quoc et al., 2012). Using DTI, we recently corroborated these results in humans with infantile strabismus in which we found that visual callosal pathways lack normal pruning (submitted). This process of elimination of juvenile visual callosal fibers thus seemed to be abnormal and to depend on post-natal visual experience. In contrast to the abundance of fibers in case of infantile strabismus, bilateral visual deprivation (as complete darkness or bilateral eyelid suture) induces fewer callosal connections (Innocenti, 1986).

The present study shows that alternative visual pathways via the posterior part of the anterior commissure, i.e., the phylogenetically and ontogenetically youngest part of the commissure (see Introduction), may compensate for the absence of callosal fibers, more specifically those that run through the splenium of the corpus callosum and connect the primary visual cortical areas, to establish binocular vision and thus depth perception. Since the anatomical accuracy of DTI is limited (Thomas et al., 2014) and because we are only looking at reconstructed fiber tracts our results do not definitely prove functional compensation. A few fibers have been described before to “project” to or to “arise” from the occipital lobe in normal subjects; interhemispheric connections through the anterior commissure have also been described before in case of agenesis of the corpus callosum (see Introduction). But this is the first time that the “primary” visual cortex is shown to be specifically involved. This is noteworthy since binocular vision and depth perception are first encoded at this level in the visual system. As an ancestral commissure (see Introduction), this indicates that the anterior commissure may replace callosal connections during pre- and/or post-natal development to ensure the same function. A similar interdependence might occur between the optic chiasm and the corpus callosum. However, both in agenesis of the corpus callosum and in chiasmal abnormalities there are multiple causes and phenotypes. The corpus callosum may on one hand compensate chiasmal decussation errors. Binocularity results in albinos show a lack of binocularity-driven cortical neurons in primary visual areas and albino binocularity is supposed to be processed through callosal connections. On the other hand, chiasmal decussation errors may also be associated with corpus callosum dysgenesis. In a case series of 9 achiasmia patients, three also had callosal agenesis and two septo-optic dysplasia. (Sami et al., 2005) Indeed, during these periods of development, the brain can easily reshape, even extensively. This is supported by previously reported data showing that transfer of visual information is highly degraded in absence of both the corpus callosum and the anterior commissure while it might appear normal when only the corpus callosum is absent with an enlarged anterior commissure (Fisher et al., 1992). Also in our case of callosal agenesis the anterior commissure appeared to be enlarged. Our data are also in line with data published by Corballis and collaborators showing that subjects with callosal agenesis may still integrate visual information (form, but not color) between the hemispheres, most likely through an enlarged anterior commissure (Corballis and Finlay, 2000; Barr and Corballis, 2002).

We only looked at one subject here. Our next step will be to extend this study to a series of patients with callosal agenesis in which the presence or absence of binocularity and anterior commissural connections is investigated. Additional data may strengthen the data we report here. Hopefully, they will also help to underline our earlier hypothesis which suggests that interhemispheric cross-talk, normally through the corpus callosum, is essential for binocular vision (ten Tusscher, 2014).

## Author contributions

Mt initiated this work. 3T GE MRI scanner Imaging and analysis of the data were done by PV, TV, Mt, AH, and Nv. 3T Philips MRI-scanner Imaging and analysis of the data were done by PV, Mt, AH, CM. Mt, Nv, AH, CM, and PV wrote the paper.

### Conflict of interest statement

The authors declare that the research was conducted in the absence of any commercial or financial relationships that could be construed as a potential conflict of interest.
